# RNase P-Mediated Sequence-Specific Cleavage of RNA by Engineered External Guide Sequences

**DOI:** 10.3390/biom5043029

**Published:** 2015-11-09

**Authors:** Merel Derksen, Vicky Mertens, Ger J.M. Pruijn

**Affiliations:** 1 Department of Biomolecular Chemistry, Institute for Molecules and Materials, Radboud University, P.O. Box 9101, Nijmegen NL-6500 HB, The Netherlands; E-Mails: M.Derksen@ncmls.ru.nl (M.D.); vicky.mertens@student.ru.nl (V.M.); 2 Department of Biomolecular Chemistry, Radboud Institute for Molecular Life Sciences, Radboud University, P.O. Box 9101, Nijmegen NL-6500 HB, The Netherlands

**Keywords:** external guide sequence, RNase P, RNA cleavage, RNA knockdown, RNA targeting

## Abstract

The RNA cleavage activity of RNase P can be employed to decrease the levels of specific RNAs and to study their function or even to eradicate pathogens. Two different technologies have been developed to use RNase P as a tool for RNA knockdown. In one of these, an external guide sequence, which mimics a tRNA precursor, a well-known natural RNase P substrate, is used to target an RNA molecule for cleavage by endogenous RNase P. Alternatively, a guide sequence can be attached to M1 RNA, the (catalytic) RNase P RNA subunit of *Escherichia coli*. The guide sequence is specific for an RNA target, which is subsequently cleaved by the bacterial M1 RNA moiety. These approaches are applicable in both bacteria and eukaryotes. In this review, we will discuss the two technologies in which RNase P is used to reduce RNA expression levels.

## 1. Introduction

For many years, strategies to interfere with gene expression post-transcriptionally have been applied to study gene function in cultured cells. Moreover, the knockdown of specific RNAs is an attractive approach for the treatment of microbial infections. Currently, multiple agents and techniques (e.g., antisense oligonucleotides and oligonucleotide analogues, ribozymes, DNAzymes and RNA interference) are available to reduce the levels of specific RNAs. In general, all these approaches involve the targeting of an RNA by an antisense nucleic acid, followed by recognition and subsequent cleavage of the duplex by an endogenous or exogenous enzyme.

**Figure 1:**
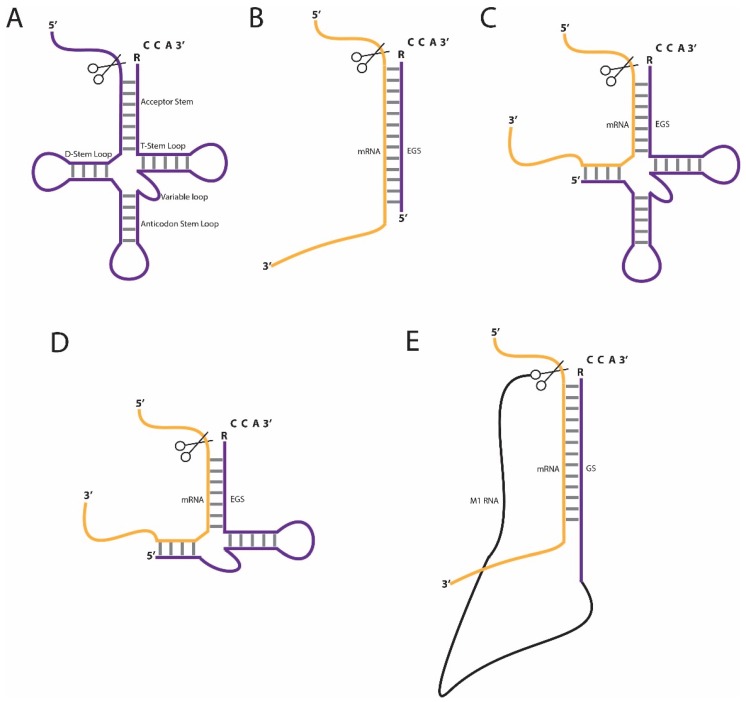
Schematic illustration of RNase P-based substrate cleavage. (**A**) Pre-tRNA, a natural substrate of RNase P; (**B**) The bacterial stem-only EGS-mRNA substrate; (**C**) The EGS-mRNA complex resembling ¾ of pre-tRNA for eukaryotic target cleavage; (**D**) The minimized eukaryotic EGS-mRNA complex and (**E**) an M1GS-mRNA complex. The RNase P cleavage activity is indicated with the scissors.

RNase P is an example of a ribozyme that can be employed for the knockdown of specific RNAs. This enzyme was first discovered in *Escherichia coli* (*E. coli*) over forty years ago and has since been identified in almost all bacteria, archaea and eukarya. The universal function of RNase P is the removal of the 5'-leader sequence from tRNA precursors (pre-tRNAs; Figure 1A) [1], but a wide range of other substrates, especially in prokaryotes, has been identified since then (reviewed in [2]).

In most organisms, RNase P is a ribonucleoprotein (RNP) complex composed of a single RNA molecule and one or more protein subunits. The RNA subunit of the RNP complexes in bacteria, archaea and eukaryotes displays catalytic activity *in vitro* in the absence of the protein subunits, which has been demonstrated for the RNA subunit from *E. coli*, *Bacillus subtilis*, methanobacteria, extreme halophiles, thermococci, *Giardia lamblia*, and humans [3,4,5]. More recently, protein-only forms of RNase P (PRORP, proteinaceous RNase P) have also been found and these act in the nucleus and/or organelles of plants, some protists, algae, as well as in animal mitochondria [6,7,8,9,10]. Interestingly, replacing the *Saccharomyces cerevisiae* RNase P RNA component by PRORP only led to minor changes in tRNA processing and, furthermore, no increase in putative RNase P substrates was observed [11].

The ability of RNase P to cleave pre-tRNA and other RNA substrates can be used to degrade specific RNA molecules. Nearly 30 years ago, Altman and coworkers found that a model substrate, which resembles the acceptor stem of a pre-tRNA, was cleaved by *E. coli* RNase P *in vitro* [12]. Soon, it was found that a bimolecular substrate composed of two complementary RNAs was also efficiently cleaved *in vitro* by the bacterial RNase P [13]. The discovery that artificial substrates can be cleaved by RNase P led to the development of different techniques in which either endogenous or exogenous RNase P is employed to knockdown specific RNAs. These techniques generally involve (external) guide sequences, which target specific RNAs and resemble the structure of the pre-tRNA substrates after base-pairing.

In this review, we will discuss the current techniques for RNA down-regulation that are based on RNase P activity. The application of these techniques will be illustrated by recent examples aimed at the down-regulation of microbial genes, genes involved in cancer and a (human) host gene that is involved in HIV1 infection.

## 2. Approaches in RNase P-Mediated RNA Knockdown

The use of RNase P in RNA downregulation is widely applicable and is often used in bacterial as well as eukaryotic cells [14,15], and to a lesser extent in plant cells [16,17]. Early applications in *E. coli* were based on an external guide sequence (EGS) that forms a stem structure with the target RNA (Figure 1B) [14,18], the endogenous RNase P recognizes this stem structure and cleaves the target RNA at the junction from single-stranded to double-stranded regions. The stem is usually between 13 and 16 base pairs long and is fused to the essential RCCA sequence at the 3'-end of the EGS, which is also present on natural pre-tRNA substrates in *E. coli*. The two cytidines in the 3'-RCCA sequence are bound by a GG sequence pair in the catalytic domain of RNase P RNA. This base pairing interaction was found to be essential for the catalytic activity in *E. coli* [19].

Unlike bacterial RNase P, the eukaryotic RNase P enzyme cannot cleave the relatively simple stem structure. Initial studies showed that in eukaryotes the EGS-target RNA duplex has to mimic 3/4 of a pre-tRNA structure, which lacks only the D-loop (Figure 1C). Subsequent *in vitro* selection studies showed that the EGS can be further minimized (Figure 1D), which results in an EGS-target RNA duplex mimicking a pre-tRNA lacking the anticodon-stem and -loop structures; furthermore, substitutions of nucleotides in the T-loop render the EGS inactive [20,21]. Since many eukaryotes produce pre-tRNAs lacking the RCCA sequence at the 3'-end, this element may not be essential for RNase P activity in eukaryotes. Indeed, it was shown both *in vitro* and in human cell cultures that the presence of the RCCA sequence on the EGS is not necessary for RNA cleavage [15,22].

While EGS technologies use the endogenous enzyme for target cleavage, in the M1 guide sequence (M1GS) approach the guide sequence is linked to the *E. coli* RNase P RNA, termed M1 (Figure 1E) [23,24]. The guide sequence forms a stem structure with the target RNA, similar to the EGS-substrate complex, and is cleaved by the conjugated M1 RNA. In *E. coli*, M1 RNA requires the C5 protein for RNase P activity, but, surprisingly, this does not seem to be the case in eukaryotes lacking the C5 protein. A major disadvantage of the M1GS compared to the EGS approach is the length of the RNA to be transfected. Compared to the M1GS RNAs, EGS RNAs are relatively short and can be easily synthesized and therefore modified to increase stability and delivery.

## 3. Stabilization and Delivery of EGS/M1GS RNAs

Due to their highly specific guide sequences, the EGS and M1GS technologies provide attractive approaches for the treatment of microbial infections, cancer and genetic diseases. However, as with other gene interference therapies, the delivery of RNAs is complicated because of multiple reasons. First, the RNA molecules may elicit innate immune responses; second, RNAs are nuclease sensitive and thus easily degraded *in vivo*; third, due to the negative charge of the RNA, the RNA molecules do not pass easily through cell membranes; and fourth, the RNAs generally need to be targeted to a specific tissue or cell type. Various efforts have been made to overcome some of the problems for *in vivo* delivery of the EGSs and M1GSs.

### 3.1. Improved Nuclease Resistance

EGS molecules can be chemically modified to make them less nuclease-sensitive [22,25,26,27]. The nucleotides in the EGS can be replaced, for example, by 2'-*O*-methyl derivatives to decrease RNase recognition and cleavage of the EGS molecules. Residues in the minimized EGS molecule, except in the loop corresponding to the T-loop of tRNA, can be substituted without loss of RNase P activity in both *in vitro* and *in vivo* studies [22,25]. In addition, phosphodiamidate morpholino oligonucleotides (PMO), which are not sensitive to cellular nucleases due to their unnatural backbone, can be used [26]. Furthermore, the addition of a 3'-3' inverted thymidine at the 3'-end of the EGS provides protection to 3'-exonucleases [25].

Besides substitution of nucleotides with 2'-*O*-methyl derivatives, oligomers composed of LNA/DNA residues can be used to improve nuclease resistance [27]. An EGS completely made up of DNA residues did not elicit RNase P cleavage *in vitro*, in contrast to co-oligomers of LNA and DNA residues. Substitution of DNA with LNA increased the thermal stability of the EGS-mRNA duplex, but the number of substitutions did not correlate with the cleavage activity *in vitro*. Interestingly, the introduction of LNA residues at the 3'-CCA and several other specific positions throughout the EGS led to high cleavage activity. These co-oligomers were also resistant to bacterial RNase treatment. Substitution of the 3'-CCA probably increases the stability of the interaction with M1 RNA, whereas the other LNA residues may improve the interaction with the target mRNA. The authors also demonstrated that these co-oligomers can reduce target mRNA levels in a permeable strain of *E. coli* at a concentration of 50 nM. Unfortunately, the DNA/LNA oligomer was not taken up by a wild type strain of *E. coli* and therefore methods to improve internalization of these molecules need to be developed.

### 3.2. EGS and M1GS Delivery

Two methods, using either conjugates of a cell-penetrating peptide (CPP) with a PMO or attenuated *Salmonella* vectors, have been developed to improve the delivery of EGS and M1GS molecules. Due to the relatively small size of EGSs, they can be easily synthesized as PMO with a modified 3'-end that can be linked to a positively charged CPP, to improve cellular uptake [28]. Altman and coworkers have shown that both the CCP and the CPP-PMO EGS directed against the bacterial *gyrA* gene, which encodes a DNA gyrase, leads to a decrease in bacterial survival. Moreover, the CPP and CPP-PMO EGS have a combined effect on bacterial death [29], although the additional effect of the CPP-PMO EGS did not appear to be very pronounced. In another study, the effects of conjugating EGSs to another CPP on target cleavage were studied. The CPP-PMO EGSs appeared to cleave target RNAs at non-predicted sites. The reasons for this miscleavage are not understood, but the positively charged peptide may interact with either the target RNA or the M1 RNA and alter the overall structure of the EGS-RNase P complex [28]. CPP-PMO EGSs have been used to kill both bacteria and malaria parasites and might become useful antibiotics [28,29,30,31].

M1GSs are approximately 400 nucleotides in length and therefore chemical synthesis will result in low yields. As a consequence, it is hard to introduce the stabilizing chemical modifications as discussed above. For the delivery of M1GSs attenuated *Salmonella* bacteria can be used. A plasmid containing a sequence encoding the M1GS is introduced into an attenuated *Salmonella* strain, which contains mutations that reduce the virulence and facilitate intracellular lysis of the bacteria and release of the transgene construct. Subsequently, this leads to efficient expression of the delivered gene in the target cells. Advantages of this system are that *Salmonella* can be targeted to specific cell types, for example macrophages, and that non-invasive oral administration is possible. This system has already been demonstrated to be successful in cell culture and mice for the delivery of both M1GSs and EGSs [32,33,34], although the mechanism by which the nucleic acid is transferred to the host remains unclear. Although not applied yet, virus-based gene delivery systems are obviously suitable as well for the introduction of M1GS molecules in target cells.

## 4. Examples of RNase P-Based RNA Knockdown

In this paragraph, we will illustrate the use of EGS and M1GS methods by describing a number of studies in which they were successfully applied in various cells and organisms to repress the levels of target RNAs. The general goal of most of these studies was to interfere with microbial proliferation and the targets can be roughly divided into four categories, bacterial, parasitic, viral and host transcripts that are involved in microbial replication. M1GSs have also been used to specifically target and inhibit growth of cancer cells. Examples of each of these categories will be discussed and are summarized in Table 1.

**Table 1:** Overview of literature data on the application of the external guide sequence (EGS) and M1GS technologies towards infectious agents and cancer.

Organism/Infectious Agent	Target Molecule	EGS/M1GS	Effect	System Tested	Reference
*E. coli*	*bla*, *cat*	EGS	Decreased viability	Cultured *E. coli*	[35]
		CPP-PMO EGS	Decreased viability	Cultured *E. coli*	[28,30]
		LNA/DNA EGS	Decreased viability	Cultured *E. coli*	[27]
	*gyrA*, *rnpA*	EGS	Decreased viability	Cultured *E. coli*	[36]
	*gyrA*	CPP-PMO EGS	Decreased viability	Cultured *E. coli* and various other bacteria	[28,29,30]
	*ftsZ*	CPP-PMO EGS	Induced filimentation and ~10-fold decreased viability ^1^	Cultured *E. coli*	[37]
*S. aureus*	*gyrA*	CPP-PMO EGS	Accelerated epithelialization and wound closure and smaller eschar formation	Cultured *S. aureus*; infected murine cutaneous wound model	[38]
*S. enterica*	*invB*/*invC*	EGS	Reduced host-invasion	*Salmonella* invasion in Henle-407 cells	[39]
*F. tularensis*	*mglB*	EGS	Reduced mRNA levels	*mglB* expression in *E. coli* culture	[40]
*Y. pestis*	*yscN*, *YscS*	EGS	Reduced mRNA levels	*yscN* and *yscS* expression in *E. coli* culture	[41]
*P. falciparum*	*PfGyrA*	CPP-PMO EGS	63%–75% growth inhibition measured in different strains	*P. falciparum* infected erythrocytes	[31]
Influenza virus	*NP*, *PB2*	EGS	70%–95% reduced expression of the viral M1 protein with the use of two EGSs simultaneously	Mouse C127 cells	[42]
Herpes simplex virus 1	*ICP4*	M1GS	1000-fold reduced virus titer (36 h, MOI 2)	ψCRE cells (NIH 3T3 cells)	[43]
	*TK*	Minimized EGS	80% decrease in mRNA and 75% decrease in protein level	Human 143tk- cells	[44]
		Minimized EGS variant, improved by *in vitro* selection	96% decrease in mRNA and 95% decrease in protein level	Human 143tk- cells	[45]
Human cytomegalo-virus	IE1, IE2	WT M1GS	500-fold reduced virus titer (5 days, MOI 1)	Human U373MG cells	[46]
		M1GS—variant G224A, G225A	3000-fold reduced virus titer (5 days, MOI 1)	Human U373MG cells	[47]
		M1GS—variant U80C, C188U	10,000-fold reduced virus titer (5 days, MOI 1	Human U373MG cells	[48]
		M1GS—variant A94G, G194C	3500-fold reduced virus titer (5 days, MOI 1)	Human U373MG cells	[49]
	AP/PR	Minimized EGS	500-fold reduced virus titer (4 days, MOI 1)	Human foreskin fibroblasts	[50]
		Minimized EGS	800-fold reduced virus titer (5 days ,MOI 2)	Human U373MG cells	[51]
		Minimized EGS variant C321	7000-fold reduced virus titer (5 days, MOI 2)	Human U373MG cells	[52]
		M1GS	100-fold reduced virus titer (5 days, MOI 3)	Human U373MG cells	[53]
		M1GS	2000-fold reduced virus titer (5 days)	Human U373MG cells	[54]
		M1GS—variant A81C, G194A	50,000-fold reduced virus titer (5 days, MOI 1)	Human U251 cells	[55]
Murine cytomegalo-virus	AP/PR	M1GS; Salmonella SL101	2500-fold reduced virus titer (4 days, MOI 1) in macrophages; prolonged survival of mice	Mouse J774 macrophages; mice	[33]
		Minimized EGS; Salmonella SL201	3000-fold reduced virus titer (4 days) in macrophages; prolonged survival of mice	Mouse J774 macrophages; mice	[34]
Hepatitis B virus	pgRNA, pre-S/L mRNA, S mRNA	Minimized EGS variant C418; Salmonella SL301	2000-fold reduced viral DNA level in HepG2.2.15 cells (3 days) and 200,000-fold reduced viral DNA level in mice (5 days)	HepG2.2.15 cells; mice	[56]
	pgRNA, pre-S/L mRNA, S mRNA	Minimized EGS variant C386; Salmonella SL201	6000-fold reduced viral DNA level (4 days)	HepG2.2.15 cells	[57]
Hepatitis C virus	HCV 5'-UTR	M1GS	>1000-fold reduced virus titer (1 day, MOI 1)	Human Huh7.5.1 cells	[58]
Human immuno-deficiency virus 1	LTR and TAT	Minimized EGS	Reduced p24 protein levels	COS cells	[59]
	TAT region	M1GS—variant G83U, G340A	150-fold reduced virus titer (12 days)	Human H9 cells	[60]
Humans	CCR5	Minimized EGS	50-fold reduced level of HIV p24 (12 days)	Human PM1 cells	[61]
	BCR-ABL	M1GS	96% and 97% cell death in respectively, p190 and p210 dependent cells	Ba/F3 cells expressing the p190 and p210 oncogenes	[62]

^1^ Decrease in viability compared to a control EGS sequence.

### 4.1. Downregulation of Bacterial RNAs and Infections

Early studies on the use of EGSs for downregulation of bacterial RNAs showed that EGSs can lead to targeted cleavage of, amongst others, the LacZ mRNA both *in vitro* and *in vivo* [14,18]. More recent research focused on the downregulation of antibiotic resistance gene transcripts by EGSs. Downregulation of these transcripts can convert antibiotic-resistant strains into antibiotic-sensitive strains and might therefore become useful tools in combatting infections with resistant bacteria. Alternatively, EGSs have been used that target bacterial genes involved in essential processes, like *gyrA*, and in virulence, like *Salmonella invB* and *invC*.

#### 4.1.1. Drug Resistance Genes

Altman and coworkers were the first to show that EGSs can be used to increase the sensitivity to antibiotics in ampicillin- and chloramphenicol-resistant *E. coli* [35]. An EGS expression system was developed in which the EGS, fused at the 3'-end to a hammerhead sequence, was expressed from a plasmid containing a T7 promoter and terminator. After transcription by T7 RNA polymerase, the hammerhead-mediated cleavage ensured the correct formation of the 3'-end of the EGS. Using this system, it was demonstrated that higher levels of the EGS as well as the expression of multiple distinct EGSs correlate with drug sensitivity. However, for obvious reasons, the delivery of a plasmid carrying the EGS into bacteria in an infected mammal will be difficult. Therefore, CPP-PMO EGSs targeting ampicillin and chloramphenicol resistance genes have been developed and these have been shown to decrease viability of ampicillin- and chloramphenicol-resistant *E. coli* [28,30]. Interestingly, reduced viability was observed both in the presence and in the absence of the antibiotics. It is likely that the reduced viability in the absence of antibiotics is due to off-target effects of the CPP-PMO EGS. Indeed, the CPP-PMO EGS targeting the chloramphenicol resistance gene appeared to be fully complementary to several *E. coli* genes. This observation implies that the capacity of the technique to eliminate bacteria extends beyond the matches with specific gene sequences. However, since the CPP-PMO EGSs seem to target multiple unintended RNAs, a major drawback of this method might be the concomitant targeting of host genes and the resulting effects on the host.

Another class of drug resistance genes are the AAC(6')-I type acetyltransferases which inactivate the antibiotic amikacin. These genes are widely disseminated and the AAC(6')-Ib enzyme especially limits the therapeutic use of amikacin [63]. Multiple EGSs were designed based on RNase H mapping of single-stranded RNA regions in the AAC(6')-Ib mRNA [64]. Subsequent *in vitro* experiments led to a selection of five EGSs based on binding affinity to the mRNA and induction of cleavage by RNase P. *In vivo* studies were performed in which the EGSs were expressed from a plasmid using the T7/hammerhead approach mentioned above [35]. EGSs with the highest binding affinities *in vitro* most efficiently decreased amikacin-resistance *in vivo*. The partial repression of amakicin-resistance, however, indicated that EGSs may aid, but may not completely eliminate infections, and therefore it has been suggested that they might be particularly useful in combination with other drugs [64]. Later, the EGS that reduced the amikacin-resistance most efficiently, was further developed to become a nuclease resistant LNA/DNA oligomer [27].

#### 4.1.2. Essential Genes

Besides drug resistance genes, two essential genes, *gyrA* and *rnpA*, have been extensively investigated as bacterial EGS targets. The *gyrA* gene encodes gyrase A, a type II topoisomerase, and *rnpA* encodes the RNase P C5 protein. Nearly 15 years ago, Mc Kinney and coworkers designed two EGSs targeting *gyrA* and two EGSs targeting *rnpA*. Expression of a single EGS targeting *gyrA* reduced *E. coli* growth approximately 7-fold, whereas the combination of both EGSs targeting *gyrA* resulted in a 10-fold reduction. The combination of all *gyrA* and *rnpA* EGSs reduced *E. coli* growth 26-fold [36]. These results confirmed the observations by Altman and coworkers that the effects of multiple EGSs on bacterial survival are additive [35]. McKinney *et al.* also showed that 1 to 3 mismatches in the EGS-mRNA duplex did not affect its inhibitory capacity, whereas five mismatches abolished this effect completely [36].

To achieve delivery into the bacteria, various CPP-PMO EGSs targeting *gyrA* have been used [28,29,30]. A CPP-PMO EGS targeting a conserved region of *gyrA* acts as a general antibiotic at micromolar concentrations for both Gram-positive and Gram-negative bacteria [29]. Since three mismatches are allowed in the EGS-mRNA duplex, it is possible to use a single *gyrA* EGS on multiple bacteria. In addition, the natural microbiome of the host might be affected. Nevertheless, it was shown that a CPP-PMO EGS targeting the *Staphylococcus aureus gyrA* mRNA led to improved wound healing of a *S. aureus* infected wound in mice [38]. Altogether, due to their effective delivery, CPP-PMO EGSs targeting essential genes are promising tools for the elimination of various bacterial infections, such as surface wound infections or internal infections, for which they can be administered orally or intravenously. Moreover, CPP-PMO EGSs may become especially useful in treating infections with antibiotic resistant bacteria.

Targeting of another bacterial gene, *ftsZ*, was investigated by Sala and coworkers [37]. *ftsZ* is the most highly conserved bacterial cell division gene coding for the proto-ring protein FtsZ, which recruits other proteins to the divisome and generates the constrictive force to initiate cell division. Because the *ftsZ* gene has no homology to eukaryotic genes, it is an attractive target for EGS-mediated downregulation. Target regions in the *E. coli ftsZ* gene were based upon the results of bioinformatic prediction of the mRNA secondary structure. Two regions were selected as good candidates, because they were predicted not to be associated with stable secondary structures. Three EGSs were designed and they all induced efficient RNase P-mediated cleavage *in vitro*. To test the activity *in vivo*, one of the EGSs was expressed using the T7/hammerhead system. This EGS showed activity in *E. coli*, because filamentation and growth impairment were observed. Nuclease-resistant EGSs that can interfere with FtsZ expression still need to be prepared. In addition, the direct delivery of these EGSs, e.g., by the conjugation to cell-penetrating peptides, needs to be optimized before they can be used to target pathogenic bacteria.

#### 4.1.3. Virulence Genes

In addition to drug resistance and essential genes, various attempts to target virulence genes by EGSs have been reported [39,40,41]. It has been shown that *Salmonella* invasion of host cells can be decreased by the downregulation of *invB* and *invC* mRNA [39]. InvB is a chaperone which is involved in actin rearrangements of the host cell and is not required for invasion; the InvC protein is an ATPase required for the type III secretion system involved in the invasion of host cells [65,66]. On the *Salmonella* genome the *invB* and *invC* genes overlap, with the last coding nucleotide of *invB* being the first coding nucleotide of *invC*. Therefore, it is likely that *invB* and *invC* are translated from a joint transcript. RNase T1 mapping of single-stranded RNA regions resulted in the design of three EGSs directed to *invC* and 1 EGS directed to *invB*. All of these induced RNase P-mediated cleavage at the predicted site *in vitro*. For *in vivo* experiments, both high- and low-copy-number plasmids were generated that allowed the production of two EGSs upon arabinose induction. These two EGSs were either both directed to *invB* or *invC*, or one targeted *invB* and the other *invC*. All plasmids were capable of decreasing the secretion of SipB and SipC proteins, which are released by the type III secretion system. As expected, the effect was less pronounced for the low-copy number plasmids (20%–30%) than for the high-copy-number plasmids (≥65%). Interestingly, in the host invasion experiments, the low-copy number plasmids were found to be incapable of reducing invasion in contrast to the high-copy-number plasmids. Since low expression of EGSs reduced type III secretion but did not detectably affect host invasion, the authors hypothesized that a certain level of inhibition of type III secretion has to be reached to inhibit host invasion [39]. In invasion studies, it was shown that a plasmid carrying two EGSs directed to *invB* also lowers secretion and invasion, which is consistent with a dicistronic *invB*/*invC* transcript.

Others have successfully reduced the levels of virulence genes of the pathogenic strains *Francisella tularensis* and *Yersinia pestis* [40,41]. In these studies, the virulence genes were expressed in *E. coli* and down-regulated with multiple EGSs and M1GSs. The *F. tularensis* virulence gene *mglB*, required for intracellular growth, was targeted with three different EGSs. After induction of expression of the EGSs, all three were capable of reducing the target mRNA level. On the contrary, three M1GSs that targeted the same sequences did not significantly down-regulate the *mglB* mRNA [40]. For *Y. pestis*, three EGSs and three M1GSs targeting the same sequence elements of the *yscN* and *YscS* mRNAs were also made. The *yscN* and *yscS* genes are required to escape from phagocytic killing by the host. The three EGSs all reduced target mRNA expression to 37%–68%, whereas only one M1GS significantly reduced the mRNA level.

Taken together, these studies showed that EGSs are effective in reducing pathogenic virulence factor mRNA levels, while the use of M1GSs has been less successful in *in vivo* studies. Therefore, EGSs targeting virulence genes are promising tools in eliminating pathogenic bacterial infections. However, in the case of *F. tularensis* and *Y. pestis*, the studies have been performed in *E. coli* and, before they can be used in therapeutic approaches, they need to be tested in the pathogenic strains.

### 4.2. Downregulation of Malaria Transcripts and Infections

The infectious disease malaria is caused by parasites of the genus *Plasmodium* and is among the most prevalent diseases worldwide. Augagneur and coworkers addressed the question whether RNase P-based modulation of gene expression could be employed to reduce *Plasmodium* proliferation [31], focusing on the genome of the *P. falciparum*, which is the most prevalent type of *Plasmodium* on the African continent. Since *P. falciparum* lacks the RNAi machinery, siRNA approaches targeting the parasitic genes are not possible. Therefore, the EGS methodology might be a feasible approach to target malaria.

A CPP-PMO EGS that targets the *P. falciparum gyrA* (*PfgyrA*) gene, which is known to be expressed in all stages of parasite development, was generated. The CPP-PMO EGS was readily detected in infected, but not uninfected erythrocytes, as well as in the parasite. *P. falciparum* infection leads to increased erythrocyte membrane permeability to a variety of substances and this may facilitate entry of the CPP-PMO EGS in the infected erythrocyte. Subsequently, the CPP-PMO EGS probably enters the parasite via a permease expressed on the parasitic membrane.

The addition of the EGS conjugate to a *P. falciparum* culture synchronized in an early developmental stage resulted in a delay in parasite proliferation, an altered morphology, as well as blocking of early development and replication. This confirmed that the *PfgyrA* gene seems to have a crucial function in parasite replication and thus is an ideal target. As the CPP-PMO EGSs were equally effective against both drug-sensitive and drug-resistant *P. falciparum* strains, these compounds may not only be used for functional analyses but also in antimalarial therapy. In addition, this technique can be used for the functional analysis of other genes of *P. falciparum* and to establish a list of alternative therapeutic targets [31].

### 4.3. Downregulation of Viral RNAs and Infections

Plehn-Dujowich and colleagues were among the first to use the EGS technology for down-regulation of viral RNAs. RNase T1 mapping was applied to select EGSs against the influenza virus *PB2* and *NP* mRNAs, which encode proteins that are involved in viral replication. They demonstrated that two EGSs targeting *PB2* and *NP* were capable of reducing the expression of the viral M1 protein in infected cells by 70%–95%, whereas a single EGS targeting *PB2* reduced M1 expression to 42%. This result shows that, like in bacteria, the use of multiple EGSs can be more efficient in inhibiting microbial growth [35,42]. Furthermore, they showed that a minimized EGS, lacking the anticodon stem and loop structures, was more effective in guiding target RNA cleavage *in vitro* as well as *in vivo* compared to the tRNA-derived EGS.

In another early application of the EGS technology the infectious herpes simplex virus 1 (HSV1) thymidine kinase (TK) mRNA was targeted. Liu and coworkers analyzed the TK mRNA structure using *in vivo* DMS mapping. A region near the translation initiation site was found to be the most accessible part of the mRNA. Stable expression of a minimized EGS targeting this region reduced the TK mRNA level by 80% in human cell lines infected with HSV1, whereas a tRNA-derived EGS reduced the mRNA level up to 50% [44]. Later the minimized EGS was further optimized by an *in vitro* selection procedure of partially randomized sequences. The resulting EGS was capable of decreasing the TK mRNA levels up to 96% in infected cells [45]. In another approach, the HSV1 *ICP4* mRNA was targeted using a M1GS [43]. The ICP4 protein is a transcription activator required for expression of the viral early and late genes [67]. Stable expression of the M1GS significantly reduced the levels of ICP4 mRNA (80%) and protein (87%) as well as that of some early and late viral genes in virus infected cells. In addition, a 1000-fold reduction of viral growth was observed.

#### 4.3.1. Cytomegalovirus

Human cytomegalovirus (HCMV) is a common herpes virus, which usually affects newborns and adults with compromised immune systems, causing significant morbidity and mortality [68]. Various efforts have been made to target and inhibit HCMV using both EGSs and M1GSs [32,33,34,47,48,49,50,51,52,53,54,55,69,70,71]. The IE1 and IE2 immediate early proteins are transcription factors involved in the activation of viral gene expression. Multiple examples are reported in which IE1 and IE2 have been targeted to inhibit HCMV proliferation [47,48,49,69]. IE1 and IE2 are encoded by the same gene and result from alternative splicing of the pre-mRNA. Liu and colleagues have used an M1GS to target the overlapping regions of the IE1 and IE2 mRNAs corresponding to a shared exon. A significant decrease in IE1/2 mRNA and protein levels, as well as the levels of some viral early and late proteins, was observed. Moreover, viral growth was inhibited 150-fold [69]. Later, two M1GS variants, which carry M1 point mutations that enhance the M1 catalytic activity, have been used to target the same region on the IE1 and IE2 mRNAs [47,48]. These variants increased viral growth inhibition compared to the wild type (WT) M1GS, leading to a 3000- and 10,000-fold reduction. In another study, an M1GS variant specifically targeting the IE2 mRNA inhibited viral growth 3500-fold [49].

Capsid assembly protein (AP) and protease (PR) mRNAs have also been extensively used as targets to inhibit HCMV growth. AP and PR are essential for viral encapsulation and have an overlapping region on their mRNAs. A minimized EGS specifically targeting the PR mRNA reduced viral growth 500-fold, whereas a WT M1GS targeting the same region seemed less effective since it reduced viral growth 100-fold [50,53]. Other minimized EGSs targeting both PR and AP have led to 800- and even 7000-fold inhibition of viral growth [51,52], whereas a WT M1GS targeting both AP and PR mRNA inhibited viral growth up to 2000-fold [54]. Very recently, an M1GS variant, carrying two M1 point mutations, was used to inhibit HCMV replication. Expression of the M1GS variant in stable human cell lines decreased the target mRNA and protein expression almost completely (99%), whereas the WT M1GS decreased the target mRNA and protein levels to 75% (mRNA determined 48 h and protein 72 h after infection). More importantly, the M1GS variant was capable to reduce viral titers 50,000-fold [55].

As discussed in the previous section, attenuated *Salmonella*-based delivery may have important advantages for therapeutic applications of guide sequences. For mouse CMV (MCMV) targeting, two strains (SL101 and SL201) were tested in cultured macrophages and in severe combined immune deficiency (SCID) mice. These strains were derived from the attenuated SL7202 strain, which is defective in aromatic amino acid biosynthesis, and in addition SL101 has a deletion of *ssrA*/*B* genes and SL201 a deletion in the *msbB* gene [33,34]. The ssrA/B and msbB genes are involved in virulence as well as pathogenesis of *Salmonella*. SL101 was used for the delivery of an M1GS construct against AP and PR. MCMV-infected macrophages treated with the SL101 *Salmonella* M1GS showed 80%–85% reduction of target protein expression and 2500-fold reduction in viral growth. Orally administered SL101 *Salmonella* M1GS in SCID mice resulted in efficient delivery to spleen and liver, as well as reduced viral gene expression, decreased viral titers, and improved survival [33]. *Salmonella* SL201 was used for the delivery of an EGS construct against MCMV PR. Treated macrophages showed a reduction of 86% in protein expression and 3000-fold reduced viral titers. In MCMV infected mice, a decrease in viral gene expression was observed, as well as viral growth reduction in various organs, and increased survival [34].

Even though *Salmonella*-based delivery of M1GSs and EGSs provides a promising approach for combatting viral infections, these *Salmonella* strains are not capable of completely eradicating the pathogens in the SCID mice, even after repeated inoculations with the M1GS and EGS strains. The reasons for this are unclear, since no research has been done to address this question. A possible explanation might be that the RNAs are not stable and rapidly degraded in the host cells. Alternatively, the effectiveness of the M1GS and EGS might not be sufficient to degrade all target RNAs in the host cell. Indeed, when using HCMV-infected J774 cells the SL101 M1GS and SL201 EGS did not completely eliminate the target RNAs [33,34]. However, in the SCID mice, the levels of target RNA were barely detectable 14 days after infection. Because M1GS variants with increased catalytic activity have been shown to be very effective in cell assays, it would be interesting to see whether *Salmonella*-based delivery of these variants could further prolong the survival of infected mice, or even completely eradicate the virus.

#### 4.3.2. Hepatitis B Virus

Hepatitis B virus (HBV) chronically infects over 400 million individuals worldwide [72]. HBV leads to a persistent and chronic life-long infection in hepatocytes, which can cause cirrhosis, liver failure, and liver cancer [73]. Delivery of therapeutic drugs specifically into the liver is a challenge. Since *Salmonella* can easily infect hepatocytes, *Salmonella* might serve as a vehicle for EGS delivery to the liver. Indeed, Lu and coworkers [57] have introduced a minimized EGS with the SL201 *Salmonella* strain into mammalian cells. The EGS was directed towards the HBV pre-genomic RNA (pgRNA) and the pre-S/L and S mRNAs. The pgRNA has a dual function as the template for viral genome synthesis and as mRNA encoding the viral polymerase and core protein. The selected target sequence also overlaps with the pre-S/L and S mRNAs, encoding viral surface antigens. The *Salmonella* delivered EGS decreased capsid associated HBV RNA 6000-fold in mammalian cells [57]. Liu and coworkers [56] used the SL7202-derived *Salmonella* strain SL301 to deliver a minimized EGS to liver cells. In the SL301 strain, the *spiR* gene, important for *Salmonella* intracellular survival and virulence, has been deleted. SL301 did not show virulence or toxicity *in vivo* as all mice were alive 85 days after inoculation. By DMS mapping experiments, the accessibility of the pgRNA in human hepatoma cells with a stable HBV infection (HepG2.2.15) was determined. *Salmonella* SL301-mediated delivery of the EGS was tested in cultured liver cells (HepG2) as well as in mice and in both cases gene transfer was successful; after 24 h, more than 70% of the HepG2 cells were infected with the SL301 carrying the EGS (SL301-EGS) and in SCID mice, a substantial amount of SL301-EGS infected cells was detected in the liver and spleen 10 days after intragastric inoculation. In HepG2.2.15, containing the full-length genome of HBV, more than 90% reduction of HBV mRNA and antigens was observed 48 h after infection with the SL301-EGS. Moreover, SL301-EGS infection led to a 2000-fold reduction in intracellular capsid-associated HBV DNA, measured 72 h after the infection. HBV cannot infect murine hepatocytes due to the lack of HBV-receptors, therefore, mice liver cells were transfected with the HBV plasmid using an intravenous hydrodynamic injection. Five days after oral inoculation of *Salmonella*, the pgRNA and pre-S/L transcripts in the liver were reduced by 95%. HBV core antigen was barely detectable in liver sections of treated mice, whereas a substantial amount of this protein was present in non-treated mice. In addition, a 95% reduction of HBV gene expression and a 200,000-fold reduction in viral DNA levels were seen in the livers and sera of mice upon treatment [56]. These results indicates that the endogenous RNase P is highly efficient and specifically cleaves target RNA, and that mutated *Salmonella* strains result in efficient delivery of the EGS transcript in the infected cells.

#### 4.3.3. Hepatitis C Virus

Hepatitis C virus (HCV) infects nearly 3% of the population worldwide and is a major cause of liver disease resulting in persistent acute and chronic infections, which can, similar to HBV, give rise to liver damage [73,74]. Currently, no vaccine is available to prevent HCV infection. The single-stranded RNA genome of HCV comprises a relatively conserved 5'-untranslated region, which contains an internal ribosome entry site (IRES) that is important for the initiation of viral polyprotein translation. Using computational secondary structure prediction, the position 67 nucleotides downstream from the first nucleotide of the HCV genomic RNA was chosen as the M1GS cleavage site, as it seemed to be in one of the most accessible regions and contains flanking sequences needed for proper M1GS interaction. Two M1GS constructs were made, M1GS-HCV/C67 with a linker sequence between the M1 RNA and the 13-nucleotide GS, and M1GS-HCV/C67* without a linker sequence. *In vitro*, the linker sequence appeared to be required for efficient cleavage of the genomic RNA of HCV [58]. To improve the pharmacokinetic properties of M1GS-HCV/C67, cholesterol was conjugated to its 5'-end, which did not diminish the cleavage activity. Both M1GS-HCV/C67 and Cholesterol-M1GS-HCV/C67 resulted in an 85% reduction in HCV core protein expression, as well as a more than 1000-fold reduction of viral growth in transfected Huh7.5.1 cells. These results indicate that the M1GS approach allows the efficient inhibition of HCV growth, that a linker between the M1 and GS parts may be crucial for activity, and that the cholesterol modification does not reduce its antiviral activity and therefore might be used to improve the kinetics for M1GS ribozymes [58].

### 4.4. Downregulation of Human Genes to Target HIV1

Human Immunodeficiency Virus (HIV) is the etiological agent of Acquired Immunodeficiency Syndrome (AIDS) [75,76]. The first study on the use of EGSs targeting HIV demonstrated that EGSs targeting *tat* mRNA and long terminal repeat (LTR) RNA can successfully inhibit HIV replication in COS cells [59]. Later, Liu and coworkers developed an M1GS-based approach against HIV1, one of the two major types of HIV, by targeting the HIV *tat* mRNA [60]. An M1GS variant, with two mutations (G83U and G340A) led to 90% reduction of viral RNA expression and a 150-fold reduction in viral growth. More recently the same researchers took a different approach by targeting the mRNA encoding the human CC-chemokine receptor 5 (CCR5) [61]. This receptor is the primary co-receptor for HIV1 to establish an initial infection and therefore an attractive host target gene to interfere with HIV1 infection. CCR5 belongs to the β subfamily of chemokine receptors and due to functional similarities within this family CCR5 was not expected to be essential for the human cell. Because a host gene is targeted, counteracting retroviral mutations are expected to occur less easily. In addition, reducing CCR5 affects the initial step of HIV1 infection before genome integration and, therefore, may prevent mutations which can cause resistance. Moreover, a naturally occurring 32 base-pair deletion in the CCR5 gene causes partial resistance towards HIV1 and therefore CCR5 was considered to be an ideal target for anti-HIV therapy.

The accessibility of the region around the translation initiation site of CCR5 mRNA was determined by *in vivo* DMS mapping and a position 29 nucleotides downstream from the translation initiation codon was chosen as cleavage site for human RNase P in the design of a minimized EGS. The CCR5 mRNA specificity of the EGS was substantiated in additional experiments. No differences in growth and viability were observed between the vector with or without EGS, indicating that the EGS does not cause significant cytotoxicity. In PM1 cells (a HIV1-permissive T-cell clone), more than 70% reduction in CCR5 mRNA and protein expression was observed, whereas control EGSs only led to minor reduction in these levels. To test the specificity and the ability of the EGS to prevent HIV infection, two HIV strains were used, the HIVBa-L strain, which depends on the CCR5 co-receptor for infection, and the HIVIIIB strain, which depends on the CXCR4 co-receptor for host infection. The EGS led to a 50-fold inhibition of HIVBa-L infection, and did not detectably affect HIVIIIB infection. Altogether, these data showed that HIV replication is inhibited by the EGS with a very high specificity. However, because 50-fold inhibition may be insufficient in completely blocking HIV infections, for therapeutic applications more efficient EGSs are required and the EGS might be combined with RNAi to enhance the efficiency of the approach [61].

### 4.5. Downregulation of the BCR-ABL Oncogenic Transcript

The Philadelphia chromosome, resulting from a translocation between chromosomes 9 and 22, is found in patients with acute lymphoblastic leukemia and chronic myelogenous leukemia. During translocation the *ABL* gene, located on chromosome 22, and the *BCR* gene, located on chromosome 9, are fused together [77,78]. The breakpoint within the *BCR* gene can be either in exon 1 or exon 3 leading to BCR-ABL fusion proteins of 190 kDa (p190) and 210 kDa (p210), respectively. These fusion proteins play a role in cancer etiology by inducing Bcl-2 mediated inhibition of apoptosis [79]. Because the chimeric mRNA resulting from the translocation is unique to the cancer cells and plays a role in cancer etiology, it is a promising target for therapeutic agents. Cobaleda and Garcia have used the M1GS approach to specifically target the chimeric mRNAs by using guide sequences targeting the *BCR*/*ABL* junctions [62]. They used murine Ba/F3 cell lines expressing either p190 or p210 to demonstrate the effectiveness and specificity of M1GSs. An M1GS targeting p190 specifically cleaved the p190 mRNA and not the p210 mRNA and *vice versa*. Moreover, treatment with the M1GS in the corresponding cell lines led to reduction of *Bcl-2* mRNA levels and enhanced cell death. In these experiments, expression of the M1GSs was achieved by stably transducing cells with a retroviral vector via electroporation. Since this delivery is not suitable for therapeutic applications, alternative delivery systems need to be explored.

### 4.6. Prophylactic vs. Therapeutic Treatment with EGS/M1GS

In the case of the bacterial targets, most of the experiments in which the effects of EGS/M1GS were assessed were performed with cultured bacteria and therefore can be considered drug-like approaches. In addition, the targeting of *S. aureus* in the murine cutaneous wound model was initiated after the infection of the wounds with the bacteria. To study the effects of EGSs on invasion of cells by *Salmonella*, EGS expression was induced in the bacteria prior to the incubation of the Henle-407 cells with the *Salmonella* strains.

The experiments that were aimed at EGS/M1GS-mediated interference of the proliferation of viruses were almost all performed in stably transfected cultured cells that expressed the EGS/M1GS prior to infection with the viruses. In some studies, the EGSs were internalized by liposome-mediated transfection procedures, but also in these cases this step preceded the infection of the cells with viruses. In spite of the prophylactic anti-viral efficacy of the EGSs and M1GSs, these molecules also appeared to display anti-viral activities in therapeutic settings. This was demonstrated in the studies with *Salmonella*-mediated delivery of the EGSs, both in cell culture and in mice. In the latter, intraperitoneal infection with the virus, for example, preceded repeated oral inoculation with the EGS-containing *Salmonella* strains. Taken together, these data strongly suggest that the EGS/M1GS approach can be applied both prophylactically and therapeutically.

## 5. EGS/M1GS Approaches *vs.* RNA Interference

The use of short RNA interference (RNAi) has also been extensively studied in eradicating microbial infections, cancer and other diseases. Currently, multiple siRNAs (small interfering RNAs) have already been or are being used in clinical trials [80]. Although EGSs and M1GSs have been successfully applied in cell cultures and mice to reduce microbial growth, to our knowledge, no clinical trials with these RNAs have been performed yet. In view of the therapeutic applicability of EGSs/M1GSs and RNAi, it would be interesting to know the difference in, for example, efficacy and potency of RNAi and EGSs/M1GSs targeting the same RNAs. However, limited experimental data is available that directly compare the use of siRNAs and EGSs [81,82]. Hayday and coworkers have targeted the thymosin beta 4 (Tβ4) gene with both short hairpin RNAs (shRNAs, which are functionally related to siRNAs) and EGSs. The results demonstrated that the efficacy of the EGS-mediated downregulation of Tβ4 mRNA was much lower (0%–15%) compared to that of shRNAs (85%–90%) [82]. In contrast, a comparison of EGSs and siRNAs targeting RNase P protein mRNAs showed that EGSs and RNAi can induce similar levels of target down-regulation [81]. Moreover, when using EGSs, a significant reduction in protein levels was already observed after 24 h, whereas it took 48 to 96 h before the effect of siRNAs became evident. It should be noted that, in both studies, tRNA-derived EGSs have been used, whereas it has been demonstrated that minimized EGSs or M1GSs can induce RNase P cleavage more efficiently. It is clear that more research is required to gain insight in the difference in efficacies of RNAi and RNase P-mediated downregulation of RNAs.

## 6. Conclusions

The RNase P EGS and M1GS technologies to knockdown specific RNAs have been shown to be a useful strategy in combating infectious diseases. EGSs and M1GSs successfully reduced bacterial viability, malaria replication and viral infections in cell cultures, and, to a lesser extent, in multicellular organisms. In general, the effectiveness still needs to be substantiated in infected organisms and also toxic side-effects need to be investigated further. *Salmonella*-based oral delivery of EGS and M1GS appears to be an attractive way to combat some viral infections in mice and possibly other organisms. Further improvements can be achieved by the introduction of stabilizing moieties and the conjugation of cell-penetrating peptides. CPP-PMOs, for example, were successfully applied in *Staphylococcus aureus* wound infections, where they improved wound healing. Although various efforts have been made to improve the targeted delivery of EGS/M1GS molecules, the delivery remains a major challenge for therapeutic applications.
